# Extracting individual neural activity recorded through splayed optical microfibers

**DOI:** 10.1117/1.NPh.5.4.045009

**Published:** 2018-12-31

**Authors:** L. Nathan Perkins, Anna Devor, Timothy J. Gardner, David A. Boas

**Affiliations:** a Boston University, Department of Biomedical Engineering, Boston, United States; b University of California, Department of Radiology, San Diego, La Jolla, United States; c University of California, Department of Neurosciences, San Diego, La Jolla, United States; d Boston University, Department of Biology, Boston, United States; e Boston University, Department of Electrical and Computer Engineering, Boston, United States

**Keywords:** imaging systems, *in vivo* imaging, fluorescence, neurophotonics

## Abstract

Previously introduced bundles of hundreds or thousands of microfibers have the potential to extend optical access to deep brain regions, sampling fluorescence activity throughout a three-dimensional volume. Each fiber has a small diameter (8  μm) and follows a path of least resistance, splaying during insertion. By superimposing the fiber sensitivity profile for each fiber, we model the interface properties for a simulated neural population. Our modeling results suggest that for small (<200) bundles of fibers, each fiber will collect fluorescence from a small number of nonoverlapping neurons near the fiber apertures. As the number of fibers increases, the bundle delivers more uniform excitation power to the region, moving to a regime where fibers collect fluorescence from more neurons and there is greater overlap between neighboring fibers. Under these conditions, it becomes feasible to apply source separation to extract individual neural contributions. In addition, we demonstrate a source separation technique particularly suited to the interface. Our modeling helps establish performance expectations for this interface and provides a framework for estimating neural contributions under a range of conditions.

## 1 Introduction

Rapid advances in genetic probes^1^ and optical techniques for recording and manipulating neural activity^2^ are central to breakthroughs in systems neuroscience, providing tools for interrogation of precisely targeted population and circuit dynamics. Yet such techniques are inherently limited by the light scattering of tissue. To gain access to more deep brain regions, researchers remove superficial tissue^3^ or employ implantable optics, such as gradient index (GRIN) lenses and prisms.^4^^–^^6^ Such implants can damage or obliterate more superficial tissue and, given dense local connectivity, may jeopardize local network dynamics in the tissue surrounding the implant.^7^^–^^9^

A similar method, fiber photometry, permits access to deep brain regions for recording bulk fluorescence via an implanted fiber-optic probe.^10^^–^^13^ The technique enables high-sensitivity recording of calcium or other fluorescent indicators expressed either in a target population or in long range axonal projections but is often limited to a single channel and requires an implant on a scale of hundreds of microns, similar to a GRIN lens. Subsequent variations have expanded the technique to record simultaneously from up to a dozen probes and/or from multiple regions.^14^ We are working to further extend the capabilities of fiber photometry, substantially increasing the number of simultaneous recording channels while decreasing the core diameter and hence the cross section of each fiber. By implanting bundles of hundreds or thousands of small diameter (<8  μm) optical microfibers, each fiber collects bulk fluorescence from a small region of tissue surrounding the aperture of the fiber while displacing substantially less tissue.^15^ During insertion, each fiber follows a path of least resistance, splaying through the target brain region. These bundles have the potential to provide a minimally invasive way to sample fluorescence activity throughout a target, nonsuperficial region of the brain.

With the capacity to record many more channels and to record more precisely localized fluorescence, we move from a paradigm of interrogating bulk activity toward one of measuring circuit-level neural encoding. Like single-photon imaging or multiunit electrophysiological recordings, it may not be possible to distinguish or limit recordings to a single neuron. But much as spike sorting enables separating individual neural activity, it is possible to incorporate knowledge of the target neural population and the sensitivity profile of the fibers to gain a more comprehensive understanding of the individual neural dynamics that will contribute to the recorded fluorescence signal.

When using a low density of fibers to interface with a low-density cell population (a small number of neurons expressing the fluorescent indicator), the sensitivity profile of each fiber will be dominated by one or two neurons. Limited overlap between the sensitivity profiles of neighboring fibers will preclude source separation, as there will not be sufficient correlations between the fluorescence signals. Alternatively, when using a higher density of fibers, the sensitivity profiles will have greater overlap, creating the opportunity to reverse the linear mixing of nearby neuronal signals. Under such conditions, the recorded signal is amenable to source separation techniques to extract individual neural traces.

In this paper, we model bundles of hundreds or thousands of optical microfibers distributed throughout a target brain region as an optical interface for collecting fluorescence activity. Our model allows us to evaluate the interface properties of the bundle and how variables like the distribution of fibers and neurons effects the interface performance. Our model results indicate that with a low density of fibers, each fiber will collect the optical equivalent of a local field potential, measuring bulk fluorescence from a small number of neurons in close proximity to the fiber tip; as the density of the fibers increases, overlap between the fiber sensitivity profiles will enable collecting sufficient information to separate individual neural activity.

## 2 Methods

### 2.1 Interface Simulation

We calculate the fluorescence sensitivity profile for a single fiber inserted into the tissue using Monte Carlo simulations of 10,000,000 photon packets launched from the fiber as described in Refs. 15 and 16. The tissue is modeled as a $1\text{-}{\mathrm{mm}}^{3}$ volume broken into isotropic 5-μm voxels, with the tip of the fiber positioned in the center of the volume. Photon packets are emitted from the fiber with an angle determined by a Gaussian distribution reflecting the numerical aperture of the fiber (0.377). The packet is modeled moving through voxels and at each step can be either scattered or fractionally absorbed. Absorption is dominated by hemoglobin in the blood, and as a result we calculate the absorption coefficient based on a 3% blood volume fraction^17^ with a 15-g/DL hemoglobin concentration^18^ and a 70% oxygenation fraction. Given the extinction coefficient of hemoglobin,^19^ our absorption coefficients are $\mu_{a}=0.337\;{\mathrm{mm}}^{-1}$ for 490-nm light, $\mu_{a}=0.343\;{\mathrm{mm}}^{-1}$ for 512-nm light. Scattering is described well in the literature, and we use a scattering coefficient of $\mu_{s}=20\;{\mathrm{mm}}^{-1}$ with anisotropy g=0.9.^20^^,^^21^ By averaging the paths of all photon packets, we construct a 3-D photon distribution throughout the tissue.

The sensitivity profile for a bundle of fibers is obtained by taking the linear superposition of the profile for a single fiber. Based on our prior histology documenting the distribution of bundles of hundreds to thousands of fibers,^15^ we estimate the distribution of fibers in the tissue as a bivariate normal distribution in xy space. To explore the interface properties under various conditions, we vary the fiber density by changing the number of fibers for a fixed standard deviation of the spatial distribution of the fibers. Unless otherwise noted, we use a standard deviation of σ=150  μm for xy splay, consistent with the observed distribution of fibers at a depth of 2 mm. The fiber depth distribution will vary based on how the fibers are cut prior to insertion; for our simulations, we model this variability as a normal distribution of depths with a standard deviation of σ=15  μm. The angle of each fiber is assumed to be uniform and parallel to the z axis.

By superimposing the sensitivity profile on the tip of each fiber in the bundle based on the splay distribution,^22^^,^^23^ we can evaluate the interface properties of the full bundle. The bundle’s ability to deliver excitation light to a given neuron is equal to the sum of all superimposed sensitivity profiles, whereas the bundle’s ability to collect fluorescent emissions from a given neuron is measured in terms of the few fibers that receive the majority of the emitted light from that neuron. The combination of the excitation and emission forms a mixing matrix

$$M_{i,j}=h_{i,j}\overset{n}{\underset{k=1}{\sum }}g_{j,k},$$ (1)

where $h_{i,j}$ is the emission from neuron j collected by fiber i and $g_{j,k}$ is the excitation from fiber k that reaches neuron j. For the analysis presented here, we assume that excitation is static—that $g_{j,k}$ does not change over time.

### 2.2 Neural Population Simulation

Equipped with this model of the optical interface properties for a bundle of microfibers, we can now evaluate the ability to measure signals from a simulated neural population. We simulate a three-dimensional (3-D) volume of tissue, of sufficient size to ensure that it includes all regions of non-negligible fluorescence sensitivity (usually around $1.2\;{\mathrm{mm}}^{3}$), with a uniform distribution of neurons. We vary the density of neurons to understand the impact of signal density, but use densities that are consistent with relevant subpopulations of interest.^24^^,^^25^ Unless explicitly stated, simulations use a density of 250,000 neurons per ${\mathrm{mm}}^{3}$. We assume all neurons express the relevant genetic probe.

Neural activity is simulated as independent spike events (Bernoulli processes).^26^ The spike probability at each time step is calculated based on the spiking frequency, which unless otherwise stated is 0.4 Hz. Spikes are then convolved with a GCaMP6 waveform; unless otherwise stated, simulations use the GCaMP6f waveform with a rise time ($t_{\text{peak}}=0.14\;s$) and an exponential decay ($t_{\frac{1}{2}}=0.32\;s$).^27^ Fluorescence was modeled at 100 time steps per second, and then downsampled based on the simulated frame rate for the recording setup.

The fluorescence traces for each neuron are then combined via the mixing matrix described in the previous section to produce fluorescence traces for each fiber. The fluorescence collected by a fiber can be written

$$y_{i}=\sum_{j=1}^{n}M_{i,j}x_{j},$$ (2)

where $x_{j}$ is the fluorescence for neuron j and $y_{i}$ is the fluorescence signal collected by fiber i.

### 2.3 Source Separation

Given the linear mixing process inherent in the fluorescence signal collected by each fiber, the data are well suited to blind source separation techniques to estimate the underlying neural fluorescence signals. To improve the performance of the source separation, we developed an approach that incorporates our knowledge of the mixing process and associated fluorescent indicator dynamics. Similar to existing techniques that use deconvolution to estimate precise spike timing,^28^ we initially inverse filter the recorded signal to remove the fluorescent indicator dynamics. This step requires an estimate of the exponential decay associated with the calcium response and indicator time constant and can be omitted for indicators where these dynamics are not known or are not stereotyped

$$Y=MX,\;\text{where}\;{\overset{\to }{x}}_{i}=\overset{\to }{w}*{\overset{\to }{s}}_{i},$$ (3)

$$Z=MS,\;\text{where}\;{\overset{\to }{z}}_{i}={\overset{\to }{w}}^{-1}*{\overset{\to }{y}}_{i},$$ (4)

where Y is the recorded fiber output, M is the mixing matrix, and X is the fluorescence signal produced by convolving the underlying signal ${\vec{s}}_{i}$ of neuron i with the indicator waveform $\vec{w}$. By applying an inverse filter (${\vec{w}}^{-1}$), we produce matrix Z that is equal to applying the mixing directly to the underlying signals.

Next, we apply the non-negative-independent component analysis algorithm^29^ to perform blind source separation under the constraints of non-negativity. The algorithm first whitens the data, and then applies a series of orthonormal rotations to reduce the error between Z and the reconstruction of Z from the rectified (non-negative) components of S.

Having separated the independent signals, we can then apply the waveform filter ($\vec{w}$) to produce fluorescence traces that correspond with the identified independent components.

As there are many more neurons than fibers, separating all neural activity is an underdetermined problem. This technique is limited to extracting as many neural traces as there are fibers.

### 2.4 Evaluating Source Separation

The evaluation of the source separation must consider both that the output of the source separation is a small subset of the underlying neural signals and the underdetermined nature of the blind source separation. To this end, we calculate the correlation coefficients of each separated signal (x→^i) with each true, simulated fluorescence trace (${\overset{\to }{x}}_{i}$). The quality of the separation is then evaluated based on how many of the separated signals have a sufficiently high correlation with a true trace. In Sec. 3, we evaluate threshold choices and how they impact the number of extracted “true” signals.

To understand the source separation in the context of an experimental question, we further evaluate the separated signals on a spike detection task. Using the approach of detecting threshold crossings as a proxy for neural spikes, we can compare threshold crossing events in the separated signal with those in the matched underlying neural trace. This forms a binary classification task that can be measured in terms of a receiver operating characteristic plot and the accompanying area under the curve (AUC).^30^ The AUC represents how well threshold crossings on the separated signal can be used to approximate threshold crossings on the true neural fluorescence; an AUC of 0.5 indicates poor performance (unable to separate), whereas a value of 1 indicates perfect performance.

As a control, we compare correlations between the separated signals with a set of randomly generated traces.

### 2.5 Code

The code to generate the fiber sensitivity profile and to run all described models are publicly available at https://codeocean.com/2018/11/12/fiber-source-separation/code. The MATLAB modeling and source separation implementation is available on Github https://github.com/nathanntg/fiber-source-separation.

## 3 Results

### 3.1 Relevant Experimental Technique

**Fig. 1:**
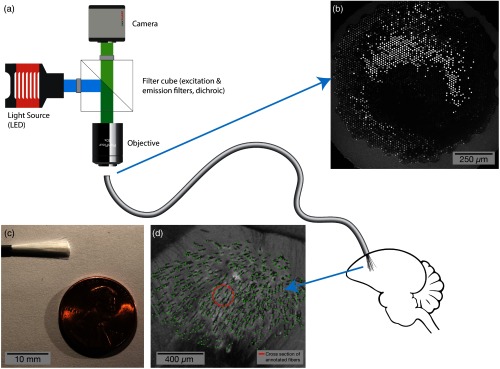
Illustration of the described method, using a bundle of optical microfibers as a multichannel, deep brain recording interface. (a) A standard fluorescent imaging configuration is used to interface with the fiber bundle. The polished imaging surface is mounted below an objective. Excitation light from an LED or other light source passes through a filter cube and is coupled into the fiber bundle; fluorescent emissions return through the objective, pass through the emission arm of the filter cube and are recorded by a camera. The fiber bundle consists of dissociated fibers, each with a diameter of 8  μm. The individual fibers are implanted into the target brain region and secured to the skull. (b) The polished imaging surface as seen by the camera. (c) A bundle of 18,000 fibers prior to implant with gray silicone sheathing cut away. (d) Histology at the tip of a bundle implanted to 2.95 mm in a zebra finch, showing 530 optical microfibers (annotated in green). The fibers displace substantially less tissue (orange circle is cross section of displaced tissue) than existing optical techniques for recording from such a large volume of tissue.

The relevant experimental technique being modeled, including the optical interface, implant methods, and motivating histology, is fully described in Ref. 15. In brief, we use commercially available flexible endoscopes (Schott 1534180), which are manufactured by dissolving an acid soluble glass between the individual fibers in coherent imaging bundles. This produces an endoscope with thousands of dissociated fibers that come together in polished imaging surfaces at both ends [Fig. 1(b)]; each fiber has a core (diameter: 5.1  μm), a cladding (thickness: 1  μm), and the remnants of the acid soluble glass (thickness: 0.4  μm). By cutting the bundle in half, the dissociated fibers are exposed [Fig. 1(c)].

After cutting away a fraction of the fibers in the bundle (based on the desired implant count), the remaining fibers are anchored together 4 to 5 mm from the tip using a bead of light-cured acrylic (Flow-It ALC, Pentron Clinical). The bead is secured to a digital manipulator in a stereotaxic rig. An anesthetized animal is mounted in the rig, and a craniotomy and durotonomy are opened above the target brain region. The fibers can then be slowly lowered into the tissue; the fibers have enough rigidity to enter the tissue without bending. Histology from implants shows that each fiber follows a path of least resistance, spreading through the target brain region [Fig. 1(d)].

The other end of the fiber (a ferrule and polished imaging surface) can be mounted below an objective in either a traditional fluorescence microscope or a purpose built optical configuration to provide excitation light and collect emission light via a CMOS sensor [Fig. 1(a)].

### 3.2 Fiber Profile Model

**Fig. 2:**
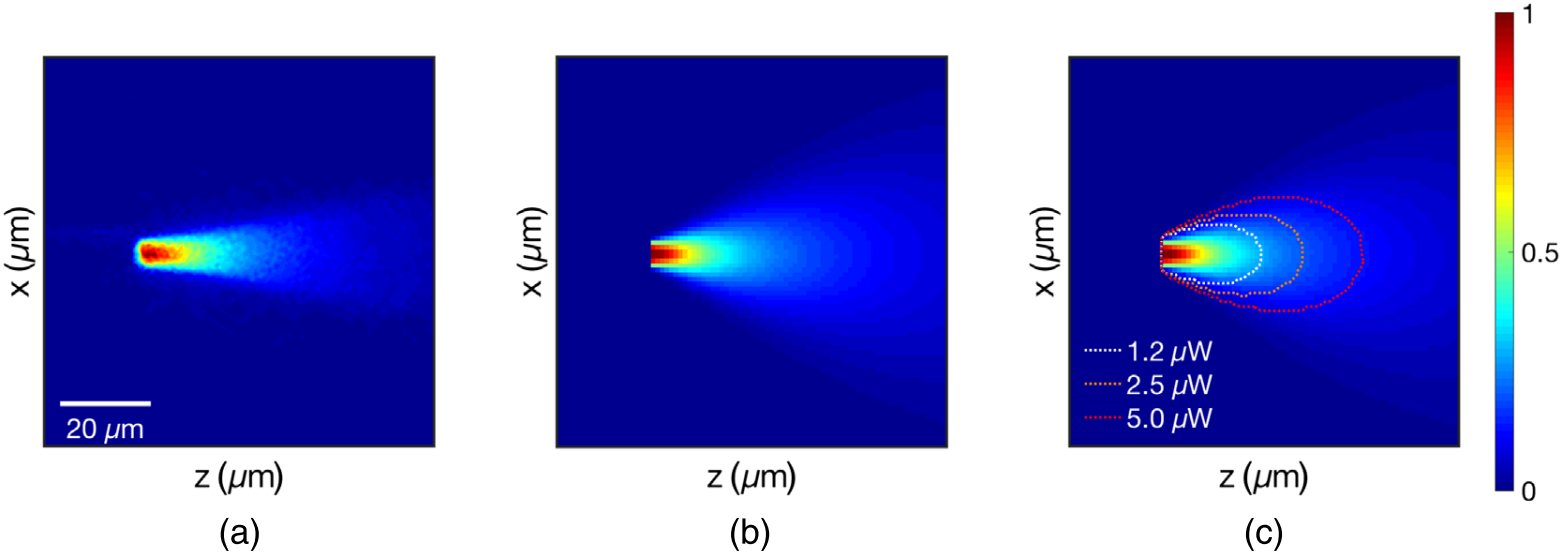
(a) A single-fiber tip in a fluorescein solution with blue light (446 to 486 nm) emitted from the fiber. Due to the near total internal reflection, the light exits the tip of the fiber. The image, captured by a fluorescence microscope with a GFP filter, reveals the fluence excitation profile for the cut fiber. Grayscale image recolored to match modeled fluorescent profiles. (b) The linear fluence excitation profile of a single fiber, calculated via a Monte Carlo simulation of photon packets propagating through water. (c) The linear fluence excitation profile from a single fiber, calculated via a Monte Carlo simulation of photon packets propagating through the tissue. At each step, a photon packet may scatter or be absorbed based on tissue properties estimated for 490-nm light. Note that incorporating tissue scattering properties does not dramatically alter the profile, as the scale is below the mean free path of light in the brain. The dotted contours show the region that will receive $2.5\;\mathrm{mW}/{\mathrm{mm}}^{2}$ or greater excitation, given coupling 1.25, 2.5, or 5  μW of light into the fiber.

In Fig. 2, we show a two-dimensional (2-D) slice of this 3-D photon distribution and compare it with a similarly generated profile of fluence in water (i.e., without negligible absorption and scattering^31^). The profile in water and tissue is similar, reflecting the fact that the relevant length scales are below the mean free path of light in brain tissue. That is, even in the tissue, photons undergo negligible scattering on the length scales relevant to the interface. In addition, we compare the two computationally generated profiles with an image of a single fiber in a fluorescein solution. Blue light is shone through the fiber, and the illuminated fluorescein is imaged through a 500- to 550-nm emission filter, revealing a distribution consistent with the model.

### 3.3 Interface Model

**Fig. 3:**
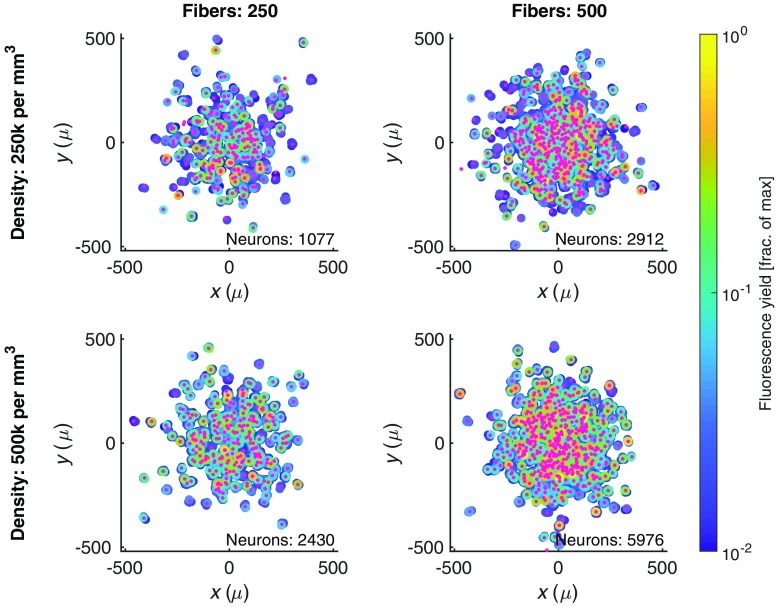
The neural population we expect to interface with through a bundle of splayed optical microfibers. The spatial distribution of fibers (small pink dots) is based on the bivariate normal distribution seen in histology slices,^15^ and is shown in relation to the neurons (large circles) that are contributing fluorescence to the collected signal above a 1% threshold. Neurons are colored based on their fluorescence signal contribution to the fiber normalized by the maximum fluorescence signal that would be recorded when a cell is immediately under a fiber. Columns show two distinct fiber counts (with same splay parameter, σ=150  μm); rows show two distinct cell densities.

Calculating the fluorescence sensitivity profiles for various distributions of optical microfibers, consistent with prior histology, we are able to model and evaluate the number and relative brightness of neurons contributing to the detected fluorescence signal. Figure 3 shows the modeled distribution of fibers and the relative brightness of individual neurons under different sets of conditions: two fiber densities (varying the number of fibers with the same standard deviation of splay) and two neural densities (reflecting different potential subpopulations). As the neural population is assumed to be uniformly distributed, increasing the density of neurons results in an increase in the number of neural traces being collected by the implant. Increasing the number of fibers has a more pronounced effect, as it increases the magnitude and uniformity of the excitation power delivered to the region and achieves a more dense sampling of fluorescence from the target neural population.

**Fig. 4:**
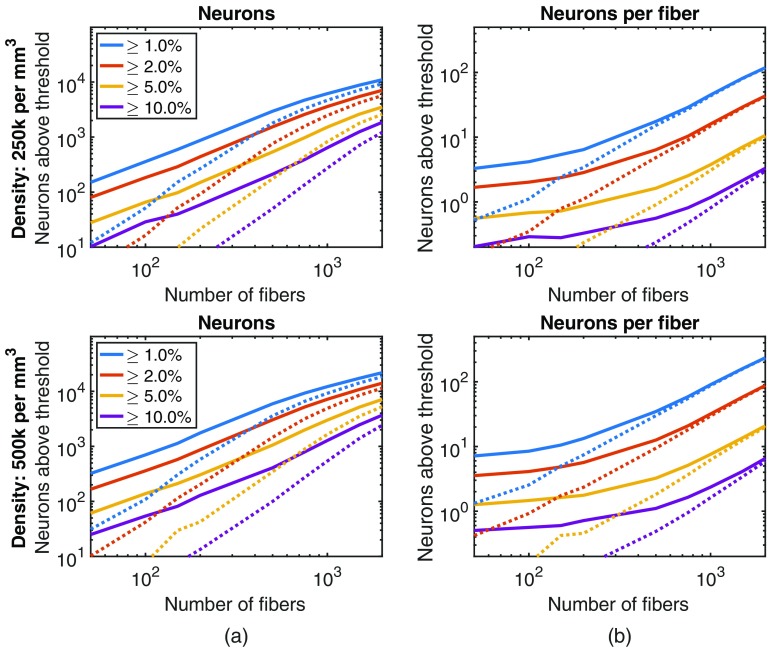
By increasing the number of fibers, while holding the splay diameter constant (σ=150  μm), the bundle can interface with more neurons. The visibility of a neuron is calculated based on their fluorescent contribution to a fiber normalized by the maximum fluorescence signal that would be recorded when a cell is immediately under a fiber. Each point is the average of five randomly generated fiber implant distributions. (a) The solid lines plot how many neurons are clearly visible (given different thresholds, represented as different color lines) to at least one fiber in the bundle. The dotted lines plot how many neurons are clearly visible to two or more fibers. (b) How many neurons are visible to a single fiber in the bundle. As the number of fibers increases, the excitation power increases and more neurons become visible.

This relationship between the number of fibers and the number of neurons contributing fluorescence is explored further in Fig. 4, with each point based on the average of five randomly generated distributions of fibers. As the number of fibers increases, we initially observe a rapid increase in the number of neurons whose fluorescence contribution to a given fiber is above illustrative thresholds, due both to the increased sampling and to the more uniformly distributed excitation power. These effects eventually saturate as the distribution of fibers is kept fixed and all neurons within the field-of-view of the fixed distribution of fibers are contributing fluorescence signals.

These results demonstrate that the number of neurons contributing to the fluorescence signal increases more rapidly than the number of fibers. Under low densities of fibers, each fiber collects from a distinct set of neurons and the low-excitation power means that the signal will be limited to the 2 to 3 neurons closest to the fiber aperture. But as the fiber density increases, the paradigm shifts to one where many neurons are contributing fluorescence to multiple nearby fibers [(Fig. 4(b)]. As multiple neural traces are present in the signal from a single fiber, it becomes beneficial to be able to apply source separation techniques.

For source separation to be effective, there needs to be an overlap in the sensitivity profiles of fibers and, as a result, neurons contributing fluorescence to multiple fibers. The dashed lines in Fig. 4 show how many neurons contribute fluorescence to two or more fibers. As the number of fibers increases, this value also increases, suggesting that source separation becomes more feasible.

**Fig. 5:**
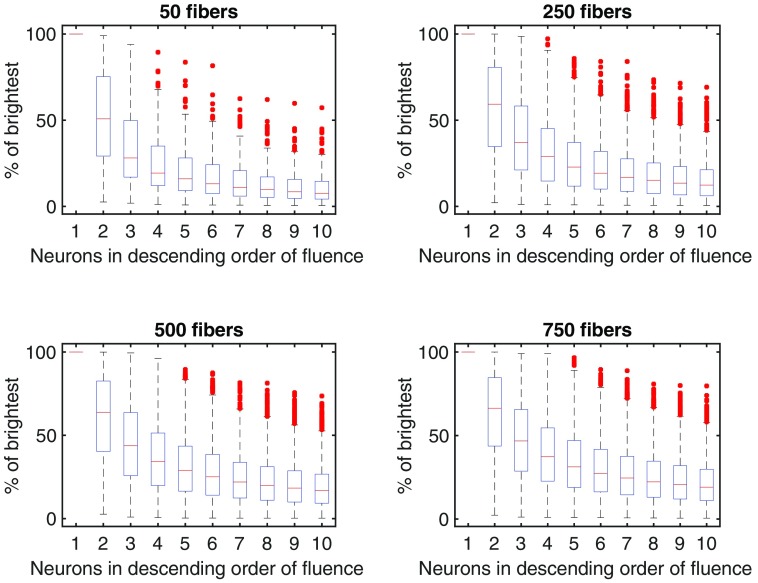
The contribution of individual neurons to the signal recorded through a single fiber. Values are normalized to the first (brightest) neuron visible to the fiber. Increasing the number of fibers does not substantially change the distribution, but delivers more light, which increases the brightness of the neurons further from the tip of the fiber. Each fiber captures fluorescence from a number of neurons, with an exponential drop off in the relative contribution of neurons. Each plot represents the average distribution of neural contributions for each fiber, averaged across fibers, and across five randomly sampled distributions of fibers. The four subfigures show increasing numbers of fibers, with a constant amount of splay (σ=150  μm).

**Fig. 6:**
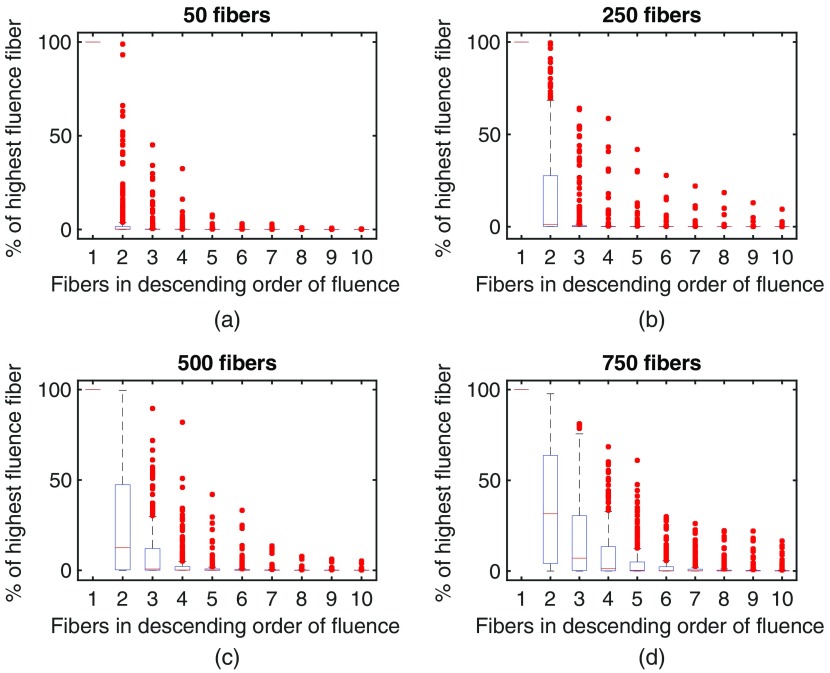
Each neuron primarily contributes fluorescence to a single fiber. Each box represents the strength of the interface between a given neuron and a fiber (round trip fluorescence), sorted in descending order of signal contributed to each fiber and normalized so that the strongest interface (the first fiber) is 100%. Plots are based on 5 sampled distributions of fibers, evaluating the 50 brightest neurons (most visible to a single fiber, in terms of round trip fluence). (a)–(d) show increasing number of fibers in a bundle, with a constant amount of splay (σ=150  μm).

The breakdown of the fluorescence signals recording by an average fiber is shown in Fig. 5, depicting the relative contribution of the neurons that most strongly interface with the fiber. Each fiber collects the fluorescence from many neurons. Figure 5 shows that the brightest cell is on average 50% to 100% brighter than the next brightest cell contributing fluorescence to a given fiber. As the density of fibers increases (and, as a result, increases the uniformity of the excitation power), the drop off in brightness from subsequent neurons in the sorted list of neurons becomes less pronounced. We can look at this same breakdown from the other perspective: how many fibers capture the fluorescence from one neuron, as shown in Fig. 6. With a low number of fibers, the overwhelming majority of fluorescence from a neuron reaches a single fiber. As the density of fibers increases though, this shifts such that multiple fibers capture the fluorescence from a single neuron, permitting the application of source separation approaches.

### 3.4 Source Separation

**Fig. 7:**
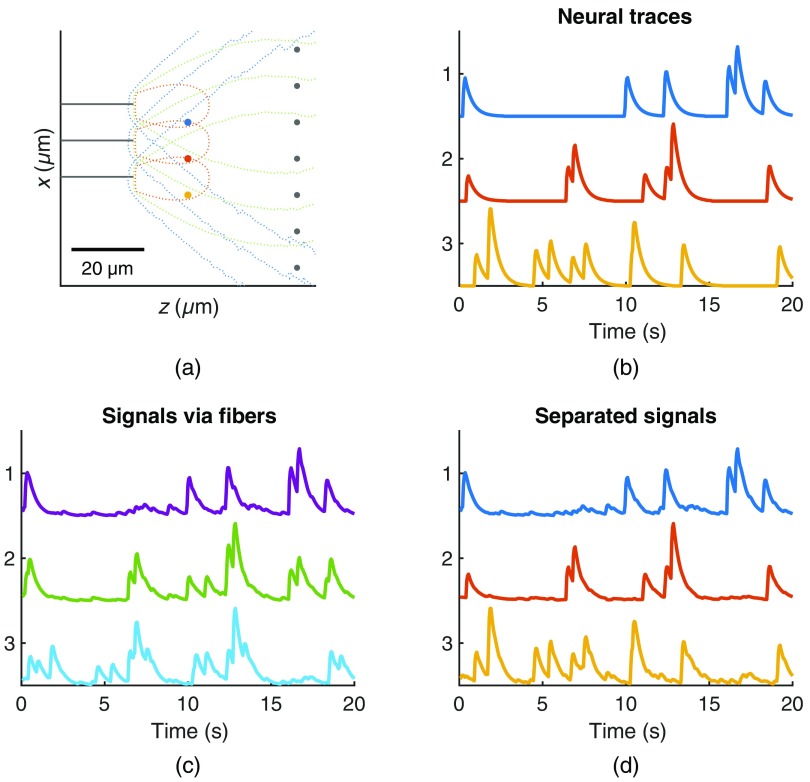
Simplified model used to exemplify the source separation process. (a) A simple 2-D configuration of fibers and neurons to demonstrate the source separation technique. Three fibers are shown in gray, each with contours representing their sensitivity profile. Three neurons (blue, red, and yellow) are positioned near the fibers while many neurons are distributed further from the fibers (creating background fluorescence). (b) Traces for the three neurons of interest generated as a random Bernoulli process convolved with a GCaMP6f waveform. (c) The signals recorded from the three fibers, representing a linear mixing of all the neurons based on the relative sensitivity. (d) The three outputs of the source separation process, sorted to most closely match the original neural traces.

Given a higher density of fibers, we can apply the blind source separation technique described in Sec. 2 to approximate individual neural components that may be contributing to the recorded fluorescence. Figure 7 shows a toy model, created to be illustrative of the linear mixing of hypothetical neural traces (based on a Bernoulli spiking process convolved with the GCaMP6f waveform) and the subsequent results of the source separation technique.

**Fig. 8:**
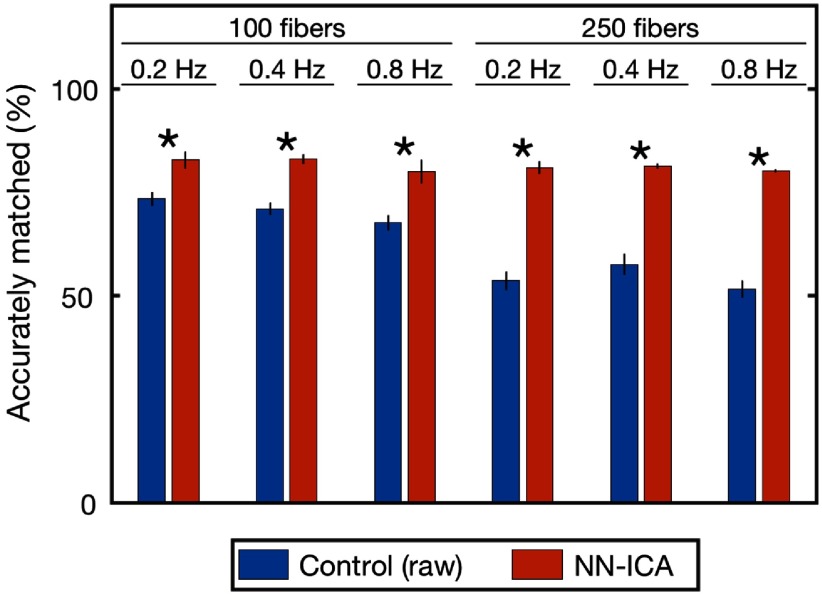
Percentage of extracted signals that accurately match an underlying neuron, based on a correlation of $r^{2}\geq 0.6$. The first column (blue) shows the accuracy of the raw fiber intensity values; as some fibers are dominated by 1 to 2 neurons, there is a high percentage of traces that closely match underlying neurons. The second column (red) demonstrates the benefit of blind source separation, increasing the number of accurately extracted signals. These results are consistent and robust across various model parameters, including number of fibers and neural firing rates. In all cases, the source separation produces a significant increase in number of accurately matched traces (paired t-test, p ≤0.01). Error bar shows std. dev. over 5 iterations.

In order to assess the performance of the source separation, the separated signals must be compared to all underlying neural activity to see if the extracted signals correspond with specific neural traces. We match extracted signals with underlying neural traces based on correlations; extracted signals having a sufficiently high correlation to the fluorescence of an underlying neuron ($r^{2}\geq 0.6$) are considered an accurate match. Figure 8 shows the percentage of extracted traces that are accurate matches with and without source separation, under a number of different model parameters (fiber counts and neural firing rate). Bundles with a larger number of fibers have a larger number of accurate matches, but calculating the percentage accuracy (normalizing by the number of fibers) better enables comparing performance across fiber counts. First, we assess accurate matches between the signals recorded via the fibers (without any source separation) and the underlying neural traces; as suggested by the earlier modeling, many fibers are dominated by 1 or 2 neurons and produce accurate matches without additional processing. By applying non-negative-independent component analysis, the number of accurate matches increases across all parameters. The increase is significant in all cases (paired t-test, p ≤0.01). The benefit of source separation becomes more pronounced with an increasing number of fibers, consistent with our expectations given the greater overlap between neighboring fibers resulting from both the number of fibers and the increased uniformity of excitation power. As a control, we compare the fiber signals with newly-generated neural traces to confirm that the identified accurate matches are not simply a probabilistic result of the large neural population being modeled; with the newly-generated data, none of the traces accurately match (not pictured), indicating that matches are not false positives.

**Fig. 9:**
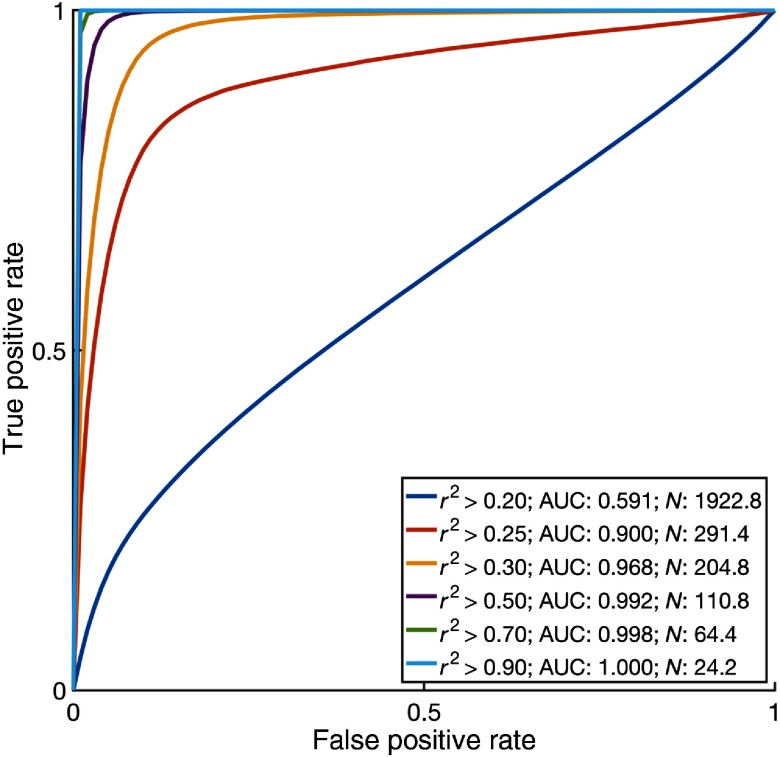
By increasing the $r^{2}$ threshold used to match separated signals with underlying neural activity, we observe an increase in accuracy in spike detection based on threshold crossing for spike detection, but a decrease in the number of matched neuronal signals. By altering the spike detection threshold, we can generate a receiver operating characteristic curve measuring the accuracy of the spike detection algorithm. Using different $r^{2}$ thresholds to match separated signals with the simulated neural fluorescence traces, we can generate different ROC curves. By lowering the $r^{2}$ threshold, we match more traces (N), but sacrifice accuracy (measured as AUC). Average of five simulations based on 100 fibers with a splay of σ=125  μm in a region with a cell density of 250,000 neurons per ${\mathrm{mm}}^{3}$.

Because this sort of evaluation may be sensitive to the $r^{2}$ threshold, we also evaluated the utility of the matched traces for different thresholds. A receiver operating characteristic curve (Fig. 9) evaluates the performance of using accurately matched, separated signals to detect action potentials (via threshold crossing) for a range of $r^{2}$ thresholds. As the $r^{2}$ accuracy threshold increases, the matched separated signals provide a more useful input for threshold detection, at the expense of reducing the number of separated signals. These results motivated the selected threshold of 0.6. As a control, the same threshold crossing detection was repeated comparing the separated signals to unrelated random traces. The AUC was between 0.5 to 0.51 for all $r^{2}$ thresholds (not pictured), consistent with the lack of a relationship between the ground truth and separated signals.

## 4 Discussion

Bundles of splaying optical microfibers present a versatile extension to existing fiber photometry methods, enabling the sampling of fluorescence from hundreds or thousands of points throughout a target brain region while displacing less tissue than traditional, large diameter photometry ferrules or GRIN lenses.

When using a small number of fibers, the method achieves a high-channel count variation of fiber photometry,^14^ collecting fluorescence activity from a small region of tissue at the tip of each fiber. As shown in Fig. 4, with a sparse target population or with sparse expression of the fluorescent indicator, this signal may correspond with just one or two cells.

As the number of implanted fibers increases or as the density of the implanted fibers increases, the recording paradigm shifts. More fibers result in greater excitation power distributed over a larger volume of tissue (and, as a result, more fluorescent signal from more neurons), and higher fiber density results in increased overlap between the sensitivity profiles of neighboring fibers. With this shift, the fluorescent signal from a single cell is more likely to contribute to multiple fibers (Fig. 5). These correlations enable application of source separation techniques to estimate individual neural activity.

Blind source separation techniques are widely used in neuroscience for decoding of neural signals,^32^ for identification of functional and anatomical connectivity,^33^ and for analyzing electroencephalography signals,^32^ as well as for applications more akin to the one described here. Source separation—specifically, independent component analysis^34^—has been applied in processing single- and multiphoton microscopy to identify and extract individual cellular contributions akin to spike sorting in electrophysiology.^35^^–^^38^

These existing blind source separation techniques can be applied directly, as the overlapping sensitivity profiles associated with each fiber act as a linear mixing of fluorescence activity from the surrounding tissue. Yet the physical distribution of the splayed optical microfibers and the mechanics of the fluorescent indicator provide useful constraints that can further inform the source separation process. Specifically, we adopt an approach that incorporates the dynamics of the fluorescent indicators and the non-negativity of the mixing process. First, we use an inverse filter to remove the fluorescence waveform. Our approach assumes consistent and known waveform dynamics, which enables the inverse filtering; should this assumption not apply, source separation techniques designed for convolutive mixtures become relevant and enable estimating both the mixing and convolution steps.^39^^,^^40^ We then use a non-negative-independent component analysis algorithm to estimate an unmixing matrix to separate the recorded signals into independent components. This achieves a linear unmixing that is consistent with the constraints of the recording mechanism, where the individual neural sources additively contribute to the recorded signals.

Similar techniques and model constraints are frequently used in traditional calcium imaging, using non-negative matrix factorization and deconvolution to extract neural activity and estimate underlying spiking.^28^ Another approach that may be applicable is Bayesian source separation, which seeks to estimate the mixing matrix and source signals by maximizing the probability of both the underlying source signals, and the resulting mixing of these source signals.^41^ Our observations about the sparseness of the mixing process, as well as any existing knowledge regarding the target population activity, can be incorporated into the prior.

In exploring source separation techniques, we also looked at what factors impact the source separation performance. Unsurprisingly, increased recording duration improves the performance. More relevantly though, source separation performance is best with sparse signals (low-spiking frequency and fast time-course fluorescent indicators, such as GCaMP6f). Such sparse signals provide discrete, differentiable events that are conducive to estimating the underlying mixing process.

To evaluate the performance of the source separation for a realistic analysis task, we compared spike detection analysis on the separated signals and on the true, underlying neural traces. Separated signals are first matched with the underlying neural traces based on correlations; the majority of the separated signals have a strong correlation ($r^{2}\geq 0.6$) with one of the true neural traces and, as a result, achieves a high level of accuracy in the threshold crossing task. Of course, analysis of real-world data would not have the benefit of being able to identify the extracted signals that match the underlying neural activity, but these results suggest that the hundreds or thousands of channels of data collected through the fiber bundle have the potential to reveal neuron-level dynamics of interest.

Further work can look at incorporating additional information into the source separation process. For example, it may be possible to estimate the initial mixing matrix through empirical measurement of correlations between fibers. Specifically, by shining light down a single fiber and looking at light collected by the other fibers, it may be possible to estimate the relative spatial configuration of the fibers and, as a result, the correlations that we would expect in the linear mixing process.

More broadly, our work here has laid a modeling framework for evaluating high-channel count fiber photometry as a means of interfacing with deep brain regions. By estimating the overlap of neighboring sensitivity profiles and hypothetical neural population dynamics, we can describe both the likely composition of the signals collected by individual fibers and the larger mixing process that occurs. One limitation of our study, although likely minor, is that we simulated neural signals as arising from point sources. Further work is needed to explore the full impact of the fluorescent signals arising from large and complex neurons, which may alter both the mixing process and the feasibility of source separation.
